# Long-Lasting Visuo-Vestibular Mismatch in Freely-Behaving Mice Reduces the Vestibulo-Ocular Reflex and Leads to Neural Changes in the Direct Vestibular Pathway

**DOI:** 10.1523/ENEURO.0290-16.2017

**Published:** 2017-02-27

**Authors:** Julie Carcaud, Filipa França de Barros, Erwin Idoux, Daniel Eugène, Lionel Reveret, Lee E. Moore, Pierre-Paul Vidal, Mathieu Beraneck

**Affiliations:** 1 Center for Neurophysics, Physiology, Pathology, CNRS UMR 8119, Université Paris Descartes, Sorbonne Paris Cité, Paris, France; 2 INRIA Grenoble, Rhône-Alpes, Laboratoire Jean Kuntzmann, UMR 5224, France; 3 Cognition and Action Group, CNRS UMR 8257, Université Paris Descartes, Sorbonne Paris Cité, Paris, France

**Keywords:** multisensory, neuronal excitability, reflex, synaptic plasticity, vestibular neurons, VOR

## Abstract

Calibration of the vestibulo-ocular reflex (VOR) depends on the presence of visual feedback. However, the cellular mechanisms associated with VOR modifications at the level of the brainstem remain largely unknown. A new protocol was designed to expose freely behaving mice to a visuo-vestibular mismatch during a 2-week period. This protocol induced a 50% reduction of the VOR. *In vivo* pharmacological experiments demonstrated that the VOR reduction depends on changes located outside the flocculus/paraflocculus complex. The cellular mechanisms associated with the VOR reduction were then studied *in vitro* on brainstem slices through a combination of vestibular afferent stimulation and patch-clamp recordings of central vestibular neurons. The evoked synaptic activity demonstrated that the efficacy of the synapses between vestibular afferents and central vestibular neurons was decreased. In addition, a long-term depression protocol failed to further decrease the synapse efficacy, suggesting that the VOR reduction might have occurred through depression-like mechanisms. Analysis of the intrinsic membrane properties of central vestibular neurons revealed that the synaptic changes were supplemented by a decrease in the spontaneous discharge and excitability of a subpopulation of neurons. Our results provide evidence that a long-lasting visuo-vestibular mismatch leads to changes in synaptic transmission and intrinsic properties of central vestibular neurons in the direct VOR pathway. Overall, these results open new avenues for future studies on visual and vestibular interactions conducted *in vivo* and *in vitro*.

## Significance Statement

Calibration of the vestibulo-ocular reflex depends on the presence of visual feedback. *In vivo* work has suggested that cerebellar-dependent calibration of VOR is, in the long-term, consolidated in the brainstem. However, the associated cellular mechanisms remain unknown. To address these mechanisms, we present an innovative protocol in which freely behaving mice are submitted to 15 d of visuo-vestibular mismatch. We demonstrated that this protocol leads to a 50% reduction of the VOR. We also showed that in brainstem slices, long-term VOR reduction is associated with synaptic and intrinsic changes within the vestibular nuclei, in the direct VOR pathway. This study opens new avenues for future studies on visual and vestibular interactions conducted both *in vivo* and *in vitro*.

## Introduction

Because of its relative simplicity, precise quantitative methods, and ease in applying experimental perturbations, gaze stabilization represents a suitable model to study motor learning that occurs when visual or vestibular sensory signals are modified (Blazquez et al., 2004; Broussard et al., 2011). In rodents, gaze stabilization depends on two complementary reflexes: the optokinetic reflex (OKR) that produces eye movements in the direction of visual motion and the vestibulo-ocular reflex (VOR) that stabilizes gaze during head motion (Stahl, 2004). Both reflexes cooperate to stabilize the visual scene on the retina in response to movement (Angelaki, 2004; Cullen, 2012).

**Fig. 1.**
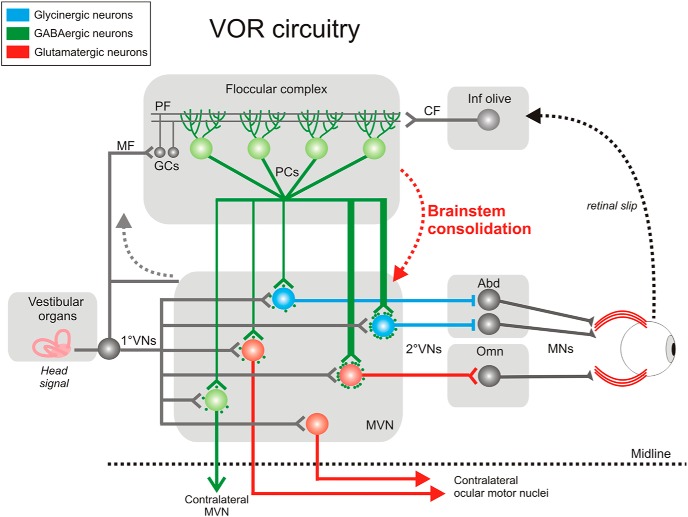
Circuitry of structures implicated in VOR and its calibration. Integration of vestibular and visual inputs in the floccular complex modulates PC outputs. Floccular target neurons in the MVN are partitioned depending on the density of innervations received from the flocculi (thin or thick lines; density of synaptic contacts), on their neurotransmitter content, and projection sites. 1°VN and 2°VN, first- and second-order vestibular neuron; MN, motoneuron; CF, MF, and PF, climbing, mossy, and parallel fiber; GC, granule cell; Abd and Omn, abducens and oculomotor nucleus.

Although the VOR operates even in the dark, its calibration depends on the presence of visual feedback. When a mismatch of visual and vestibular information occurs, retinal slip results, which blurs vision. This retinal slip serves as an error signal that is conveyed to the inferior olive and then to the cerebellum, where it is integrated with other sensory inputs (Fig. 1). The error signal indicates that eye movements are not compensatory, and thus drives motor learning to modulate the VOR (Shin et al., 2014) and restore gaze stabilization, a process known as VOR adaptation. The role of the cerebellum in the induction and short-term retention of this motor learning is clearly established (Hansel et al., 2001; Boyden et al., 2004; Carey, 2011; De Zeeuw and Ten Brinke, 2015). In contrast, long-term retention of the memory in the cerebellum itself or its transfer to downstream structures has long been a topic of debate (Broussard and Kassardjian, 2004; Broussard et al., 2011). One hypothesis was that the cerebellum is the main site of memory retention, as demonstrated by long-term depression at the synapses between parallel fibers and Purkinje cells (Fig. 1; Marr, 1969; Albus, 1971; Ito, 1982). An alternative model proposed that the cerebellum would not be the only site of retention but would also provide a teaching signal guiding the induction of plasticity within the brainstem (Miles and Lisberger, 1981; Lisberger et al., 1984). In favor of this hypothesis, experiments using cerebellum deactivation demonstrated that flocculi shutdown suppresses VOR short-term, but not long-term, adaptation (Luebke and Robinson, 1994; Pastor et al., 1994; Nagao and Kitazawa, 2003; Kassardjian et al., 2005). The retention of oculomotor memories outside the cerebellum in the long-term was further confirmed by OKR experiments (Shutoh et al., 2006; Anzai et al., 2010; Okamoto et al., 2011).

Although the hypothesis of a consolidation of long-term VOR changes in the brainstem has received support from many theoretical studies (Porrill and Dean, 2007; Masuda and Amari, 2008; Menzies et al., 2010; Medina, 2011; Clopath et al., 2014; Yamazaki et al., 2015), it has little experimental support. Studies *in vivo* have shown that some vestibular nuclei neurons changed their activity after VOR adaptation (Keller and Precht, 1979; Lisberger, 1988; Lisberger et al., 1994), even though this effect could not be dissociated from Purkinje cell activity. It was also proposed that the modification of the strength of the synapse between vestibular afferents and central vestibular neurons could be a key mechanism involved in VOR calibration (Menzies et al., 2010; Yamazaki et al., 2015).

Many of the studies on VOR motor learning have been conducted on animal models that do not allow for *in vitro* investigation. On the other hand, the use of the mouse model has its own constraints, as long-term modification of the VOR is classically achieved through passive head-fixed, iterated discontinuous training sessions interrupted by intertrial intervals of variable duration (Boyden and Raymond, 2003). Here, the neural basis of VOR plasticity was evaluated with a new long-term VOR reduction procedure in mice. Using a combination of behavioral analyses, oculomotor measurements with or without floccular deactivation, and *in vitro* electrophysiological recordings, we provide evidence that long-term VOR reduction is accompanied by synaptic and intrinsic modifications in the direct VOR pathway.

## Material and Methods

### Animals and surgical procedures

All animal procedures were performed in accordance with the University Paris Descartes animal care committee’s regulations. A total of 116 C57BL/6J male mice (Janvier Labs; RRID: IMSR_JAX:000664) aged 6–8 weeks were included in the protocol. Gas anesthesia was induced using isoflurane. The head was shaved using an electric razor, and a 2-cm longitudinal incision of the skin was made to expose the skull. A small custom-built head holder (0.3 × 0.3 × 0.5 mm) was fixed to the skull just anterior to the lambda landmark using dental cement (C&B Metabond; Parkell). After the surgery, animals were isolated and closely monitored for 48 h. Buprenorphine (0.05 mg/kg) was provided for postoperative analgesia, and care was taken to avoid hypothermia and dehydration.

### Visuo-vestibular mismatch protocol

**Fig. 2.**
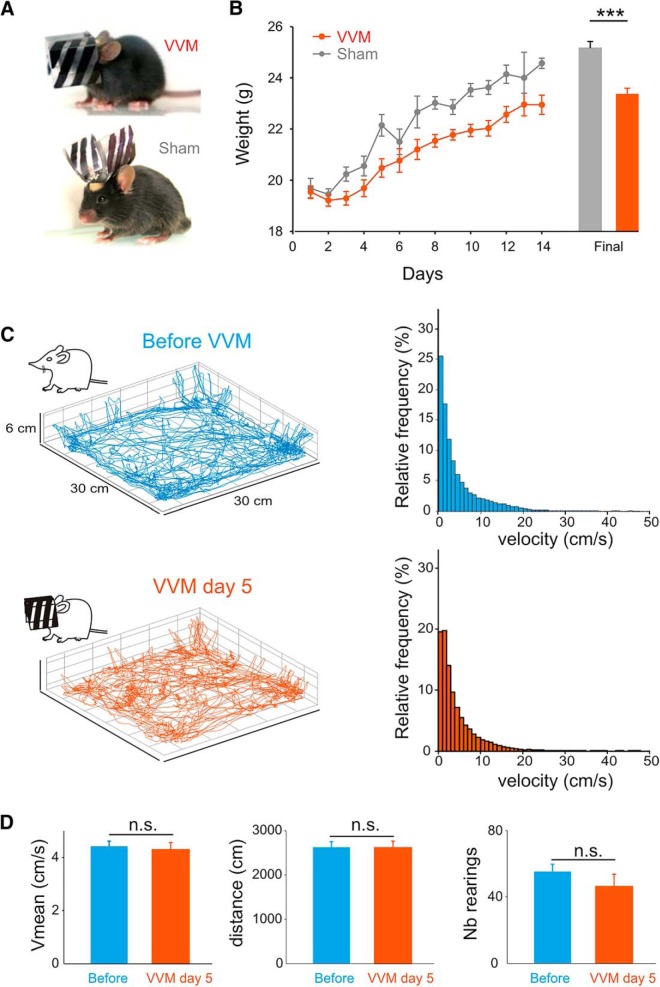
VVM protocol and open-field experiments. ***A***, Pictures of a mouse during VVM (top) or sham (bottom) protocols. ***B***, Mean body weight of sham (*n* = 24, gray line) and VVM mice (*n* = 57, red) during the 2 weeks of the protocol. ***C***, Locomotion of mice before VVM (top) or after 5 d of VVM protocol (bottom) recorded while the animal explores the open field. Left panels, examples of 3D reconstruction of the path of the same animal. Right panels, distribution of velocities for the population of mice (*n* = 4) before VVM (blue) or after 5 d of VVM (red). ***D***, Plots of the mean velocity (Vmean, in cm/s), total covered distance (in cm), and vertical explorations (number of rearings) of mice (*n* = 4) before and after 5 d of VVM. Error bars represent ± SEM.

Two days after the surgery, a custom-built device was secured on top of the head holder. The device consisted of a helmet (2.2 cm width × 1.5 cm depth × 1.5 cm length; weight 2 g) that completely covered the mouse’s head. The front of the device was adapted to the mouse anatomy so that the nose was not covered, and its width allowed for grooming and barbering behaviors. To preserve light-dependent physiology and nychthemeral rhythm, the device was made of nonopaque plastic with a thickness of 0.3 mm. In addition, 3-mm large vertical black stripes were drawn on the external surface to produce a high-contrast head-fixed visual signal during self-generated movements (Fig. 2*A*, top). A similar procedure was used on sham animals, except that the device was attached upside-down so that it did not cover the face of the mouse (Fig. 2*A*, bottom). Therefore, sham animals were exposed to the same surgical procedures and wore the same device but did not experience the visual-vestibular mismatch. Visuo-vestibular mismatch (VVM) and sham animals were housed together. Animals were housed in groups of three to stimulate social interactions. After 2 weeks with the device on the head, mice were immediately tested in behavioral experiments or used for *in vitro* electrophysiological experiments. The VOR of mice used for electrophysiology measures was not recorded to avoid relearning/extinction processes.

### General observations

During the first hours of the VVM protocol, mice displayed disturbed behavior including difficulties orienting in the cage, bumping into walls, and reduction in social interactions. Initial difficulties to access food and drink were also noted, and animals therefore received intensive attention during the initial 48 h of the protocol. This early period corresponded to a decrease in the weight of the animal (Fig. 2*B*). On the other hand, sham mice did not show any sign of disturbed behavior after beginning the protocol. After 2 d, the general behavior of VVM mice returned to normal, with good orientation in the cage, normal locomotion, and social interactions. The increase in weight observed thereafter was comparable to that of sham animals; still, after 2 weeks, a significant weight difference of ∼1.5 g persisted between the two groups (Mann–Whitney, *z* = 4.28, *p* = 0.00002).

### Behavioral measures

#### Open-field test

To determine how the VVM affects the way mice move, 3D tracking during open-field exploratory behavior was performed before the beginning of the protocol and 5 d after the device was implanted, i.e., once general behavior was back to normal. The setup consisted of a 30-cm cube surrounded by eight CCD cameras (synchronization frequency: 10 Hz; Point Grey Research, GRAS-03K2M). Mice were placed, one at a time, at the center of the arena and left completely undisturbed for 10 min. The 3D trajectory of the center of volume of the mouse was recovered using a multiple-view optical system. The images were electronically synchronized at the frame level using a trigger signal and a time stamp. For each camera, a calibration procedure provided the geometric projection from a 3D reference frame to the 2D image plane using a pinhole model. The 3D reference frame was oriented so that the *XY*-plane corresponds to the ground plane (*Z* = 0) and the *z*-axis to the up vertical axis. For each image, a background subtraction technique isolated the 2D silhouette of the mouse. The centroid of the silhouette provided a 2D cue of the animal’s center of mass. After calibration of the cameras, the 2D centroids of each frame were triangulated using a direct linear transformation technique to compute the 3D trajectory of the center of volume. The third vertical coordinate was used to count the number of rearings. This experiment allowed analysis of the whole-body velocity, total distance traveled, and number of rearings performed during exploration of the open field.

#### Video-oculography and vestibulo-ocular performance

Video-oculography was performed to quantify the gaze-stabilizing reflexes. Mice were head-fixed at a ∼30° nose-down position to align the horizontal canals in the yaw plane (Calabrese and Hullar, 2006; Oommen and Stahl, 2008) and placed in a custom-built Plexiglas tube secured on the superstructure of a vestibular stimulator. VOR performance was tested before and after the VVM protocol, with all sources of light turned off except for the computer screen. The turntable was further surrounded with a closed black box to isolate the animal from any remaining light, with an intensity inside the box <0.02 lux. Ten minutes before the experiment, 2% pilocarpine was applied to the eye to keep the pupil size constant (van Alphen et al., 2010). To record the effect of the VVM on the VOR, the head-fixed animal was put on the vestibular turntable immediately after the removal of the device while kept in the dark to avoid visual access in the environment.

Eye movements were recorded using an infrared video system (ETL-200, Iscan). The video-oculography calibration procedure was similar to that described by Stahl (2004). Eye and head position signals were sampled at 1 kHz, digitally recorded (CED power1401 MkII) with Spike 2 software, and later exported to Matlab for offline analysis (Matlab, The MathWorks; RRID: SCR:001622). Horizontal eye and head movement data were digitally low pass-filtered (cutoff frequency: 40 Hz), and position data were differentiated to obtain velocity traces. Segments of data with saccades were excluded from VOR slow-phase analysis. For horizontal sinusoidal rotations, at least 20 cycles were analyzed for each frequency. VOR gain and phase were determined by least-squares optimization:

$$EH_{v}\left(t\right)=g\times \left\{\left[HH_{v}\times \left(t-t_{d}\right)\right]+C^{te}\right\},$$

where *EH_v_*(*t*) is eye horizontal velocity, *g* (gain) is constant value, *HH_v_* is head horizontal velocity, *t_d_* is the dynamic lag time (in ms) of the eye movement with respect to the head movement, and *C^te^* is an offset. *t_d_* was used to calculate the corresponding phase φ° of eye velocity relative to head velocity. The variance-accounted-for (VAF) of each fit was computed as

$$1-\left[\frac{\mathrm{var}\left(est-EHv\right)}{\mathrm{var}\left(EHv\right)}\right],$$

where var represents variance, *est* represents the modeled eye velocity, and *EHv* represents the actual eye horizontal velocity. VAF values were typically between 0.70 and 1 (∼97% of recordings), where a VAF of 1 indicates a perfect fit to the data. Trials for which the VAF was <0.5 were excluded from the analysis.

Overall, the VVM protocol was tested on 25 mice: *n* = 13 for the characterization of VOR features and *n* = 12 for VOR dependency on rotation frequency. Horizontal VOR in the dark was first tested during sinusoidal angular rotation around the vertical axis (0.5 Hz; velocity in range 20–50°/s) on 13 mice after VVM and on six sham mice. The ratio of slow-phase reduction was calculated by dividing the post-VVM measure by the pre-VVM measure. To compare how VVM affects slow phases versus quick phases, quick-phase analysis was performed at the highest tested velocity (0.5 Hz; 50°/s). A total of 20–25 cycles were analyzed in 10 of 13 VVM animals that had a VOR reduction >60%. The number of quick phases was counted, and their amplitudes were analyzed in Matlab.

To further characterize the slow-phase reduction, the dependence on the stimulation frequency on 12 additional VVM and six sham mice were tested at different frequencies of 0.2, 0.5, 1, and 2 Hz and at a fixed peak velocity of 30°/s.

#### Optokinetic reflex performance and flocculi shutdown experiments

To test whether the VOR reduction depends on the cerebellum or on a different brain structure, a flocculus shutdown experiment was performed. During these tests, the OKR was recorded to validate the effect of the pharmacological inhibition of the flocculus/paraflocculus complex. To record the OKR, the mouse was surrounded by a 40-cm-wide dome, and sources of light were turned off except for the optokinetic projector. The light intensity inside the dome during OKR testing was measured at 185 lux (Luxmeter Lux-1337 Iso-tech). The optokinetic full-field stimulation was performed by projecting a dot pattern at velocities of 7.5°/s in both clockwise and counterclockwise directions. The dot pattern consisted of 25,000 white dots (max width 0.075°) randomly distributed on a black background. Optokinetic constant velocity stimulations lasted 1 min and were separated by at least 2 min of darkness. Optokinetic responses were analyzed offline after being imported into Matlab. Segments of data with saccades were excluded from the analysis. Optokinetic responses were measured as the mean eye velocities on segments longer than 1 s. Optokinetic gains were then calculated as the ratios of the mean eye velocities to the constant drum velocity.

After 2 weeks of VVM, both OKR and VOR performances were tested on seven mice. OKR and VOR were again measured 30 min after the stereotaxic injection of lidocaine (*n* = 5; 1 μL, 2% lidocaine) or sham injections of vehicle (*n* = 2) in the bilateral flocculi following a medial (3 mm medio-lateral) dorso-ventral direct approach (6 mm antero-posterior; *The Mouse Brain in Stereotaxic Coordinates*, Paxinos and Franklin) as in Shutoh et al. (2006). Injection was performed under isoflurane anesthesia to allow a rapid recovery and eye movement measurements. VOR testing was performed after OKR measures in complete darkness at frequencies of 0.2, 0.5, 1, and 2 Hz and at a fixed peak velocity of 30°/s, as described above.

To confirm the injection was made in the flocculi, fluorescent dye (1% fluorescein isothiocyanate, Invitrogen) was added to the lidocaine or saline injection and visualized using an epifluorescent microscope on subsequently cut slices, following the procedure described by Okamoto et al. (2011). After intracerebellar injections, intracardiac perfusions were performed, and the brains were removed. The brains were postfixed overnight in PFA 4% and dehydrated in 30% sucrose solution for at least 48 h. Then, 80-μm brain slices were cut using a microtome and visualized with the epifluorescent microscope (Olympus BX-61).

### Electrophysiological experiments

#### Whole-cell patch-clamp recordings

Brain dissections and patch-clamp recordings were performed on slices taken from control (*n* = 36) or VVM (*n* = 38) animals. After decapitation under deep anesthesia (pentobarbital 100 mg/kg), the brain was quickly removed and placed in ice-cold, phosphate/bicarbonate-buffered artiﬁcial cerebrospinal ﬂuid (ACSF), which included (in mm) 240 sucrose, 2.5 KCl, 1 NaH_2_PO_4_, 25 NaHCO_3_, 3 MgCl_2_, and 10 glucose and was supplemented with 95% O_2_-5% CO_2_. Brainstem slices of 220 µm containing the medial vestibular nuclei (MVN) were cut using a microslicer (Leica). To optimize the maintenance of vestibular afferent fibers in the plane of the slice, an angle of 15° was added to the standard coronal plane. Slices were then transferred into an incubating vial ﬁlled with regular ACSF containing (in mm) 120 NaCl, 2.5 KCl, 1 NaH_2_PO_4_, 25 NaHCO_3_, 2.5 CaCl_2_, 2 MgCl_2_, and 10 glucose and oxygenated with 95% O_2_-5% CO_2_ (pH 7.4). The more rostral slices containing the MVN, in which the brainstem was attached to the cerebellum (5.8–6.5 mm AP; *The Mouse Brain in Stereotaxic Coordinates*, Paxinos and Franklin), were selected and placed in the recording chamber maintained at 32–34°C. Slices were superfused with regular ACSF at a constant ﬂow rate of 3 ml/min. Patch-clamp pipettes were pulled from borosilicate glass tubing to a resistance of 4–7 MΩ. The internal solution (Sekirnjak and du Lac, 2006) contained (in mm) 140 K-gluconate, 2 MgCl_2_, 5 KCl, 10 HEPES, 0.03 CaCl_2_, 0.1 EGTA, 4 Na_2_ATP, and 0.4 Na_2_GTP (adjusted to pH 7.3 with KOH).

MVN neurons were visualized with a microscope (Olympus BX-51) using differential interference contrast illumination with Nomarski optics. Using the boundaries of the fourth ventricle as landmarks, MVN neuron recordings were restricted to the dorsal half of the nucleus (depth of 4–4.25 mm) and to the medial two thirds of the nucleus (0.25–0.8 mm lateral; *The Mouse Brain in Stereotaxic Coordinates*, Paxinos and Franklin). Neurons at the edge of the fourth ventricle were not recorded. After the surface of the soma of a neuron was approached with a pipette, suction was applied until a giga-ohm seal was made. Recordings were made with a Multiclamp 700B (Molecular Devices). Inhibitory transmission through glycine and GABA_A_ receptors was blocked using 10 µm strychnine and 100 µm picrotoxin, respectively (Sigma-Aldrich; McElvain et al., 2010). The spontaneous discharge was first recorded in current-clamp mode for few minutes until a stable level was reached. MVN neurons that had a membrane potential less than –45 mV and a spike amplitude >45 mV were selected for current and voltage clamp experiments. The current and voltage from the amplifier were low-pass filtered at 2 kHz and digitized at 5 kHz (BNC-2090 + PCI-6052E, National Instruments). Custom-written codes in Matlab were used for acquisition and offline analysis.

#### Excitatory postsynaptic current and plasticity recordings

Excitatory postsynaptic current (EPSC) recordings were performed on brain slices cut from 12 control mice (*n* = 17 neurons; 13 from 8 naive mice and 4 neurons from 4 sham mice) and 19 VVM animals (*n* = 31 neurons). After recording the spontaneous discharge and action potentials at the basic membrane potential, the neuron was clamped at –70 mV. First, the vestibular afferents were stimulated with a concentric bipolar stimulating electrode (FHC) placed on the vestibular fiber bundle. Electrode placement was always made at the same optimal location 4.5 mm ventral to the horizontal plane and ∼1.7 mm lateral (*The Mouse Brain in Stereotaxic Coordinates*, Paxinos and Franklin), similar to the procedure followed by McElvain et al. (2010). EPSC were evoked (eEPSC) using stimulation at a frequency of 0.067 Hz (one stimulation every 15 s). First, the stimulation amplitude was set at 300–400 pA to maximize the EPSC amplitude. Then, after 3 min of recording, a long-term depression (LTD) protocol consisting of 30 repetitions of 550-ms vestibular nerve stimulation at 100 Hz was applied according to the procedure provided by McElvain et al. (2010).

All analyses were performed offline in Matlab. eEPSC amplitude (pA), area under the curve (AUC, fC), and time constant (τ, ms) were calculated offline. For the LTD protocol, eEPSCs were normalized to the preprotocol baseline, and the mean value collected during the 15 min after the LTD protocol was calculated for graphic representation.

#### Electrophysiological properties of MVN neurons

Basic and firing properties of MVN neurons were investigated in current-clamp (*n* = 63 control neurons from 36 mice and *n* = 60 VVM neurons from 38 mice). Because most MVN neurons are spontaneously active in slices, the potential was low-pass filtered at 1 Hz to obtain an estimate of its average resting level that was taken as the “mean average membrane potential” (Vm, in mV) of each neuron. This membrane potential value was corrected offline by measuring and subtracting the extracellular voltage offset found after withdrawal of the electrode from each neuron. Averages of the spike shapes and following interspike interval profiles were analyzed to obtain the spontaneous firing rate (in spikes/s), the associated coefficient of variation (CV), the amplitude of the afterhyperpolarization (AHP, in mV), the spike threshold potential (in mV), the concavity, and the convexity (in mV). The AHP and interspike interval first derivative was used to quantify the amplitude of the double AHP (dAHP, in V/s) and the presence of an A-like rectification (AHPR in V/s). The classification of MVN neurons was performed based on these quantitative criteria following the procedure provided by Beraneck et al. (2003). Excitability of the neurons was tested using injection of hyperpolarizing/depolarizing steps from basic potential with steps of currents (1 s; 25pA increment, range ± 125 pA). Excitability was calculated as the mean firing frequency during the steps (in spikes/s). Membrane resistance was calculated from the hyperpolarizing step at –75 pA.

### Statistical analyses

Statistical analyses were performed using Statistica 7.1 software (StatSoft). A statistical table is provided (see Table 1). Repeated-measures ANOVAs were performed on VOR gain and phase across frequencies or velocities. Nonparametric unpaired Mann–Whitney tests were performed to compare measures between control and VVM mice. Nonparametric paired Wilcoxon signed rank tests were performed to compare EPSC amplitude before and after the LTD protocol.

**Table 1.** Statistical table.

Fig.	Panel	Data structure	Type of test	*p* value
2	B	Non-normal distribution	Mann–Whitney test	0.00002
2	D left (Vmean)	Non-normal distribution	Wilcoxon test	0.46
2	D middle (distance)	Non-normal distribution	Wilcoxon test	0.14
2	D right (nb rearings)	Non-normal distribution	Wilcoxon test	0.14
3	C left (gain)	Normal distribution	ANOVA repeated measures: group effect	<0.0001
3	C left inset (gain)	Normal distribution	ANOVA repeated measures: group effect	0.046
3	C right (phase)	Normal distribution	ANOVA repeated measures: interaction group x velocity	0.022
3	C right inset (phase)	Normal distribution	ANOVA repeated measures: group effect	0.96
3	D left (occurrence)	Normal distribution	paired *t*-test	0.004
3	D right (amplitude)	Normal distribution	paired *t*-test	0.11
3	E	Correlation	Pearson correlation significance test	0.0003
3	G left (gain)	Normal distribution	ANOVA repeated measures: frequency effect	0.36
3	G right (phase)	Normal distribution	ANOVA repeated measures: frequency effect	0.002
4	A/before vs. VVM	Non-normal distribution	Wilcoxon test	0.62
4	A/VVM vs. lidocaine	Non-normal distribution	Wilcoxon test	0.001
4	A/VVM vs. sham	Non-normal distribution	Wilcoxon test	0.68
4	C left (gain)	Normal distribution	ANOVA repeated measures: before vs. after group effect	0.017
4	C left (gain)	Normal distribution	ANOVA repeated measures: after vs. shutdown group effect	0.28
4	C right (phase)	Normal distribution	ANOVA repeated measures: after vs. shutdown group effect	0.49
5	B left (AUC)	Non-normal distribution	Mann–Whitney test	0.0018
5	B middle (tau)	Non-normal distribution	Mann–Whitney test	0.26
5	B right (amplitude)	Non-normal distribution	Mann–Whitney test	0.004
5	C control	Non-normal distribution	Wilcoxon test	0.017
5	C VVM	Non-normal distribution	Wilcoxon test	0.33
6	B left (all neurons)	Normal distribution	ANOVA repeated measures: group effect	0.316
6	B middle (type A)	Normal distribution	ANOVA repeated measures: group effect	0.0004
6	B right (type B)	Normal distribution	ANOVA repeated measures: group effect	0.88

## Results

A new protocol was developed to expose freely behaving mice to a VVM for a 2-week period. The consequences of the VVM on locomotor behavior and its effects on the efficacy of the VOR were first tested. Next, the neural changes underlying long-term VOR reduction were studied using whole-cell patch-clamp electrophysiology on brainstem slices.

### Behavioral experiments

#### Open-field data

During the freely behaving VVM protocol, the animal generates active movements. To determine whether the implanted device modifies the way the animal moves, the exploratory behavior of four mice was analyzed using a 3D tracking video system. When placed in the open field, mice naturally explored the environment in both horizontal and vertical planes (Fig. 2*C*, left). Exploratory behavior was compared for each mouse before the beginning the protocol (blue trace) and 5 d after the device was implanted (red trace), while the mouse was still wearing the device. Overall, we observed no difference in the way the mice explored the open field. The distribution and range of walking velocities were comparable (Fig. 2*C*, right), and there was no difference in the mean velocity (*n* = 4, Wilcoxon test, *z* = 0.73, *p* = 0.46), total covered distance (*n* = 4, Wilcoxon test, *z* = 1.46, *p* = 0.14), or number of vertical explorations (rearings; *n* = 4, Wilcoxon test, *z* = 1.46, *p* = 0.14; Fig. 2*D*). These data complement our general observations (see Materials and methods; Fig. 2*B*), which suggest that after the initial 48 h, mice wearing the VVM device display relatively normal behavior.

#### VVM protocol reduces the VOR

**Fig. 3.**
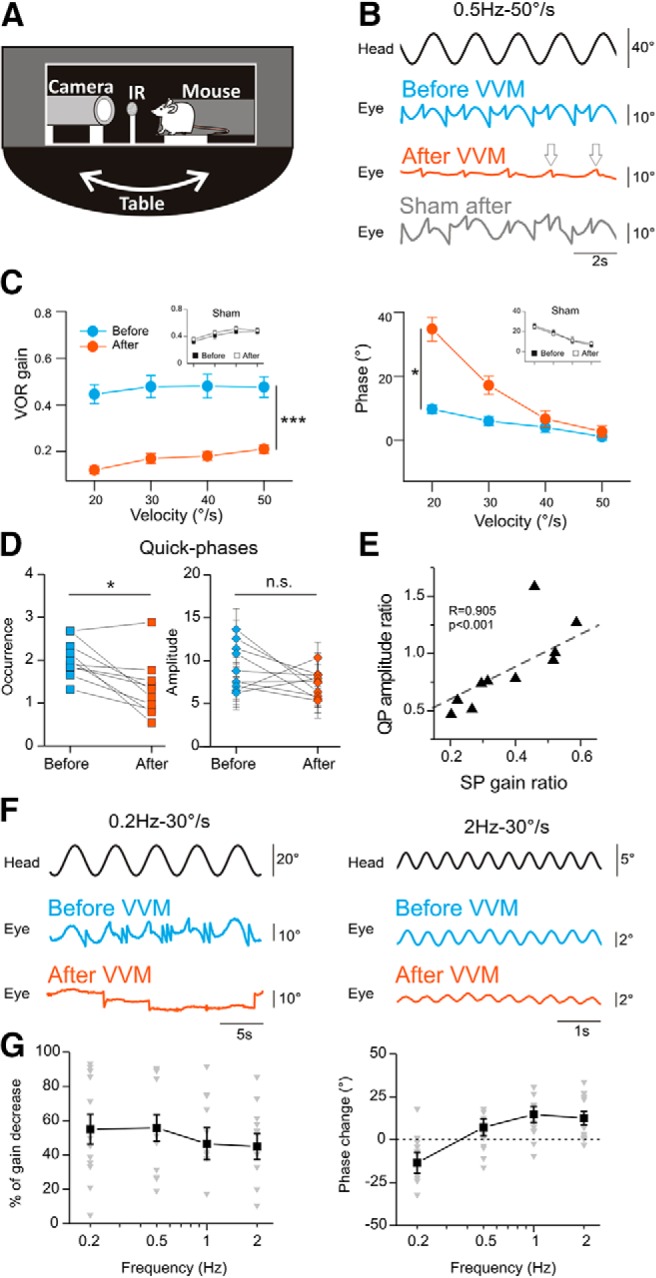
VOR reduction. ***A***, Illustration of the setup used to test the VOR. IR, infrared light. ***B***, Example raw traces of the VOR in the dark recorded before (blue line) and after 2 weeks of VVM (red line) from the same animal. Gray trace, sham mouse tested after 2 weeks of wearing the helmet. White arrows indicate example of quick phases observed after VVM. Head and eye traces show rightward movements in the upward and downward directions, respectively. ***C***, Mean VOR gain (left) and corresponding phase (right) plotted as a function of tested velocity (*n* = 13 mice; fixed frequency of 0.5 Hz) measured before (blue lines) and after 2 weeks of VVM (red lines). Insets: sham (*n* = 6 mice) before (filled squares) and after (empty squares) the protocol. ***D***, Occurrence (left) and amplitude (right) of quick phases (*n* = 10 mice). ***E***, Quick-phase amplitude ratio (after/before values) is significantly correlated to slow-phase gain ratio (***p < 0.001). ***F***, Example raw traces of the VOR reduction at 0.2 or 2Hz stimulation. ***G***, Mean percentage of gain decrease (left, *n* = 12) or phase change (right, *n* = 12) depending on stimulating frequencies. The gray triangles represent the individual values, and the black lines represent the mean values. Error bars represent ± SEM.

To evaluate the effects of the VVM on gaze stabilization, the VOR was quantified using video-oculography in the dark during sinusoidal rotations around a vertical axis (Fig. 3*A*). The VOR of 25 mice was compared before the implantation of the VVM device and immediately after its removal 2 weeks later; it was similarly measured on six sham mice. As a result of VVM, the amplitude of the eye movements observed during table rotations was greatly reduced (Fig. 3*B*, response of the same mouse before and after VVM). A first group of 13 mice was tested at a fixed frequency of 0.5 Hz and peak velocities from 20 to 50°/s. A decrease of the VOR gain by >50% was observed for all conditions after the 2 weeks of VVM (Fig. 3*C*, left, repeated-measures ANOVA, group effect, *F*_1,24_ = 42.6, *p* < 0.0001), with a shift toward phase lead at velocities <40°/s (Fig. 3*C*, right, repeated-measures ANOVA, group × velocity interaction effect, *F*_3,72_ = 3.41, *p* = 0.022). In contrast, sham mice (*n* = 6) exposed to a comparable experience in the absence of VVM had no reduction in VOR (slight increase in VOR gain: repeated-measures ANOVA, group effect, *F*_1,5_ = 6.92, *p* = 0.046; VOR phase: repeated-measures ANOVA, group effect, *F*_1,5_ = 0;003, *p* = 0.96; see traces in Fig. 3*B*, *C*). In addition to the slow-phase changes, quick phases were also investigated. After VVM, the occurrence (Fig. 3*D*, left) of the quick phases was significantly diminished (*n* = 10, paired *t*-test, *t* = 3.77, *p* = 0.004) and although there was a tendency of decreased amplitude of saccades after VVM, this difference did not reach statistical significance (Fig. 3*D*, right; *n* = 10, paired *t*-test, *t* = 1.77, *p* = 0.11). However, the comparison of the changes in slow phases and quick phases demonstrated a significant correlation (Fig. 3*E*; *R* = 0.905; *z* = 1.50, *p* = 0.0003), suggesting that both features of the vestibulo-ocular reflex were modified by the VVM.

Because the VVM was generated by an actively behaving mouse, we then questioned how this reduction affects the VOR across different frequencies. The dependence of the VOR reduction on the stimulating frequency was therefore tested on the six sham animals and 12 additional VVM mice at four different frequencies (0.2, 0.5, 1, and 2 Hz; fixed peak velocity of 30°/s). No differences were found in the VOR gain and phase of sham animals before and after the protocol (mean gain ± SD before/after: 0.2 Hz, 0.14 ± 0.05/0.16 ± 0.06; 0.5 Hz, 0.34 ± 0.05/0.32 ± 0.08; 1 Hz, 0.50 ± 0.09/0.55 ± 0.1; 2 Hz, 0.59 ± 0.18/0.55 ± 0.13; repeated-measures ANOVA, group effect; for VOR gain: *F*_1,5_ = 0.007, *p* = 0.94; for VOR phase: *F*_1,5_ = 0.99, *p* = 0.36). Fig. 3*F* shows examples traces of the VOR generated at frequencies of 0.2 Hz (left) or 2 Hz (right) before and after VVM. Again, we found a significant decrease of the gain of the VOR at all frequencies after VVM, represented by a percentage of gain decrease of ∼50%. Although the average decrease of the VOR was comparable at all frequencies (repeated-measures ANOVA, frequency effect, *F*_3,33_ = 1.11, *p* = 0.36), Fig. 3*G* illustrates the variability in the amount of VOR decrease between individuals (gray triangles). The long-term VVM also had a significant effect on the phase of the VOR, with a shift toward greater phase lag at 0.2 Hz and toward greater phase lead at frequencies ≥0.5 Hz (Fig. 3*G*, right, repeated-measures ANOVA, frequency effect, *F*_3,33_ = 6.30, *p* = 0.002). Overall, video-oculography results clearly demonstrate that the 2 weeks of VVM protocol led to a strong gain-down reduction of the VOR.

#### Flocculus shutdown experiment

**Fig. 4.**
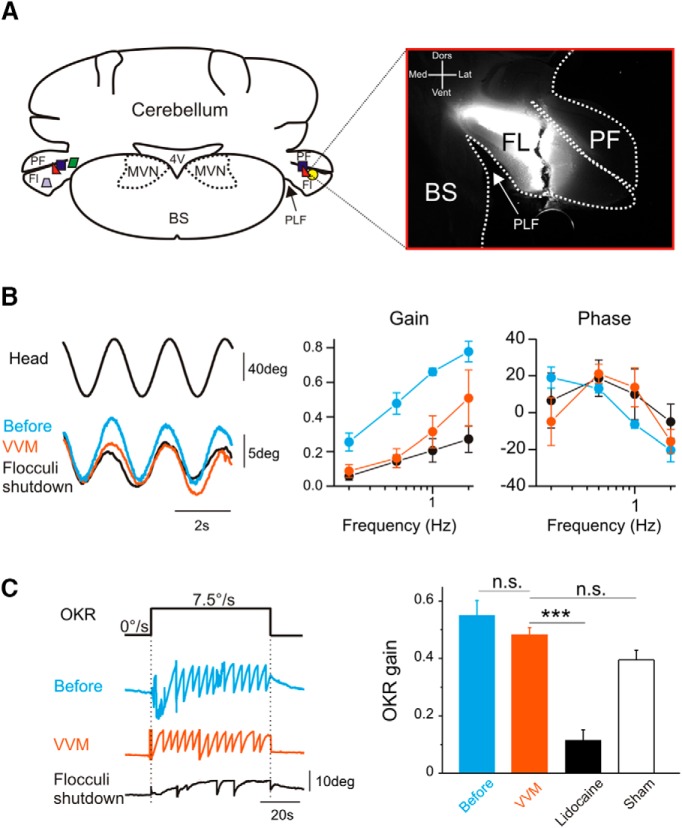
Flocculi shutdown experiments. ***A***, Left, coronal section of the brainstem and cerebellum illustrating the lidocaine injections in flocculi complex. PF, paraflocculus; Fl, flocculus; PLF, posterolateral fissure; 4V, 4th ventricle; BS, brainstem. Right, example of a lidocaine injection coupled to fluorescein isothiocyanate. Dors, dorsal; Vent, ventral; Med, medial; Lat, lateral. ***B***, Left, example raw traces of VOR in the dark recorded before (blue line), after 2 weeks of VVM (red line), and after flocculi shutdown (black line). All traces are from the same animal. Right, Bode plots of VOR gain and phase (*n* = 5 mice). ***C***, Left, example raw traces of eye movements recorded during optokinetic stimulation (60s-long full-field stimulation at 7.5°/s constant velocity). All traces are from the same animal. Right, mean OKR gain recorded before, after VVM, after lidocaine injection, or on sham animals. Error bars represent ± SEM.

To determine whether the long-term VOR reduction depends on cellular changes located at the level of the cerebellum or in downstream structures, flocculus shutdown experiments were performed by injection of lidocaine (Fig. 4). Injections of lidocaine were coupled to fluorescent dye to validate *a posteriori* the location in the flocculi (Fig. 4*A*). As described above, VOR gain significantly decreased after 2 weeks of VVM (Fig. 4*B*, *n* = 5; repeated-measures ANOVA, before vs. after group effect, *F*_1,6_ = 10.52, *p* = 0.017). When the flocculi were inactivated, the gain and phase of the VOR remained unchanged compared to the values reached after VVM (Fig. 4*B*; repeated-measures ANOVA, after vs. flocculi shutdown group effect, on gain measures: *F*_1,8_ = 1.35, *p* = 0.28; on phase measures: *F*_1,8_ = 0.52, *p* = 0.49). To functionally confirm the efficacy of the injection, the OKR was measured on the seven mice before and after VVM, and immediately after injection of lidocaine (*n* = 5) or saline (*n* = 2). Before VVM, optokinetic full-field stimulation performed at 7.5°/s triggered a consistent response (Fig. 4*C*, blue trace). After 2 weeks of VVM, optokinetic responses were mostly preserved (Fig. 4*C*, red trace; Wilcoxon test, *p* = 0.625). We noted, however, that OKR responses were sometimes qualitatively less robust than before VVM (not shown). After lidocaine injection in the flocculi, the OKR responses were largely abolished (Fig. 4*C*, black trace), as demonstrated by the strong reduction of the mean gain after the lidocaine injection (Wilcoxon test, *p* < 0.001). As expected, sham injection did not significantly modify the OKR responses (*n* = 2, 0.38 ± 0.13, Wilcoxon test, *p* = 0.686). The OKR tests therefore confirmed the ability of lidocaine injection to inhibit the flocculi. Overall, these experiments demonstrated that after 2 weeks of VVM, the reduction of the VOR depends on plasticity located outside the flocculus/paraflocculus complex.

### *In vitro* electrophysiological recordings

To determine whether the VOR reduction involves plastic changes at the level of the brainstem, *in vitro* electrophysiology was performed on mice after the 2 weeks of VVM and compared with control mice. Previous studies suggested that the reduction of the VOR could depend on plastic changes in the direct horizontal VOR pathway. Therefore, we measured the synaptic and intrinsic properties of central MVN neurons after VOR reduction.

#### Synaptic plasticity after VVM protocol

**Fig. 5.**
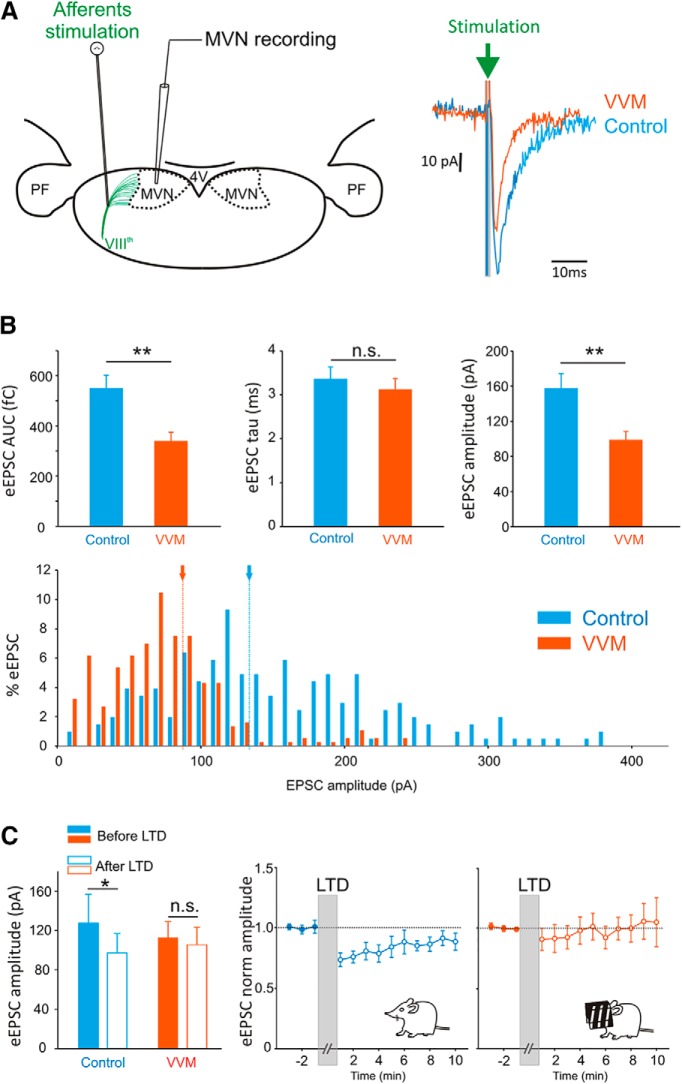
Stimulation of afferents: vestibular synapses efficacy. ***A***, Left, illustration of *in vitro* patch-clamp recordings of MVN neurons on coronal brainstem slice. The stimulating electrode is placed on the central vestibular fiber bundles. PF, parafloccular region; 4V, 4th ventricle. Right, example raw traces of superimposed eEPSC recorded from a MVN neuron of a control mouse (blue line) and from a mouse after VVM (red line). ***B***, Top, evoked EPSCs AUC (in fC), time constant (τ, in ms), and amplitude (in pA) recorded from control neurons (*n* = 17) or after VVM (*n* = 31). Bottom, distribution of eEPSC amplitude for control (blue bars) and VVM (red bars) conditions. Arrows and dashed lines indicate the medians (∼135 pA for control and ∼85 pA for VVM). ***C***, Plasticity of vestibular nerve synapses onto MVN neurons. Left, eEPSC amplitude recorded on control (*n* = 8) and VVM (*n* = 14) neurons before (filled bars) and after (empty bars) LTD protocol. Right, mean eEPSC peak amplitude before and after LTD protocol for control (blue line) and VVM (red line) neurons. The eEPSC values are normalized to the mean baseline value before LTD protocol (filled circles). Empty circles represent measures following LTD protocol. Error bars represent ± SEM.

Whole-cell patch-clamp electrophysiology was performed on central neurons recorded in brainstem slices taken from control and VVM mice. We first recorded evoked eEPSCs from second-order neurons in response to afferent stimulation (vestibular afferent fiber bundle stimulation; Fig. 5*A*, left) in control (*n* = 17 neurons) and VVM (*n* = 31 neurons) conditions. As illustrated in Fig. 5*A*, right, eEPSCs were smaller in VVM neurons compared with control neurons, a result confirmed by an AUC (Fig. 5*B*, top left) that was significantly smaller in the VVM than in control condition (Mann–Whitney test, *z* = 3.13, *p* = 0.0018). To determine whether this decrease in eEPSC AUC depends on changes in postsynaptic receptors, we explored the kinetic characteristics of the eEPSC. The eEPSC time constant (τ) was not different between the two groups (Fig. 5*B*, top middle, Mann–Whitney test, *z* = –1.12, *p* = 0.26). In contrast, the amplitude was smaller after VVM in comparison with control mice (Fig. 5*B*, top right, Mann–Whitney test, *z* = 2.84, *p* = 0.004), as shown in the distribution of eEPSC amplitude shifted toward smaller amplitudes (Fig. 5*B*, bottom). These results suggest that the receptor units involved in the eEPSC responses are qualitatively not different in VVM mice from controls. Altogether, this experiment suggests that the long-term VOR reduction is associated with a reduction of the efficacy of the synapses between vestibular afferents and central vestibular neurons.

Last, we asked whether additional down-tuning of this synapse was possible or whether the synaptic efficacy was already at its minimum. To address this question, we performed a LTD protocol in both control and VVM mice (Fig. 5*C*). As previously reported (McElvain et al., 2010), LTD could be experimentally induced in control slices, which led to a significant decrease in the eEPSC amplitude after the stimulation protocol (*n* = 8; Wilcoxon test, *z* = 2.38, *p* = 0.017). On the other hand, when neurons from VVM mice were tested, no further decrease of the synapse efficacy could be triggered (*n* = 14; Wilcoxon test, *z* = 0.97, *p* = 0.33).

Overall, these data show that the reduction in the VOR observed after VVM is correlated with a reduction in the efficacy of the synapses between the vestibular nerve and central vestibular neurons. Because no additional decrease of the synaptic efficacy could be triggered experimentally, we then explored whether these synaptic changes were supplemented by a change in the intrinsic excitability of central vestibular neurons.

#### Intrinsic properties of MVN neurons after VVM

To explore this hypothesis, we first characterized the membrane properties of the neurons recorded in slices of control and VVM mice (Table 2). Because vestibular neurons have a pacemaker activity in brainstem slices (Dutia et al., 1992, 1995), we first characterized their responses in the absence of external stimulation (Table 2). There was no difference in the spike shape parameters between control and VVM conditions (*n* = 63 control neurons vs. *n* = 60 VVM neurons). However, VVM neurons showed a decrease of their spontaneous firing rate in comparison to control neurons (Mann–Whitney test, *z* = –2.49, *p* = 0.013) and a slightly more regular spontaneous discharge than control neurons (CV of 0.16 vs. 0.22; Mann–Whitney test, *z* = 1.97, *p* = 0.048). MVN neurons were previously shown to be composed of different subpopulations that can be partly segregated using their electrophysiological signature at rest (Straka et al., 2005; Eugène et al., 2011; Beraneck and Idoux, 2012). Hence, we further divided the recorded neurons using the canonical type A and type B classification. This analysis revealed that compared with controls, type A neurons appear to be specifically modified by the VVM, with a tendency for a lower firing rate than control neurons (Mann–Whitney test, *z* = –1.73, *p* = 0.08).

**Table 2.** Static intrinsic properties of MVN neurons.

	All		type A		type B		Statistical tests (Mann–Whitney)					
Property	Control	VVM	Control	VVM	Control	VVM	Control vs. VVM					
Number of neurons	63	60	27	17	36	43	All		type A		type B	
Membrane potential (mV)	–48.28	–49.00	–47.24	–47.22	–49.07	–49.70	*z* = –1.09	NS	*z* = –0.02	NS	*z* = –0.81	NS
Spike threshold (mV)	–31.32	–32.34	–29.58	–30.73	–32.63	–32.97	*z* = –1.36	NS	*z* = –0.99	NS	*z* = –0.39	NS
Firing rate (Hz)	12.24	7.85	13.83	8.14	11.04	7.74	*z* = –2.49	*p* = 0.013	*z* = –1.73	*p* = 0.08	*z* = –1.57	NS
Coefficient of variation	0.16	0.22	0.15	0.22	0.17	0.22	*z* = 1.97	*p* = 0.048	*z* = 1.40	NS	*z* = 1.11	NS
AHPR (V/s)	0.20	0.21	0.44	0.67	0.02	0.03	*z* = –1.29	NS	*z* = –0.09	NS	*z* = –0.11	NS
dAHP (V/s)	0.71	0.69	0.00	0.00	1.24	0.96	*z* = –0.07	NS	NA	NA	*z* = –1.14	NS
AHP (mV)	26.11	25.25	29.68	28.54	23.43	23.95	*z* = –0.16	NS	*z* = –0.14	NS	*z* = 0.48	NS
Concavity (mV)	–1.33	–1.50	–2.49	–3.47	–0.46	–0.73	*z* = 0.002	NS	*z* = –1.49	NS	*z* = –0.59	NS
Convexity (mV)	0.82	0.84	0.56	0.34	1.02	1.05	*z* = 0.13	NS	*z* = –0.98	NS	*z* = 0.03	NS

Parameters of the spontaneous activity of MVN neurons recorded on control and VVM slices. Static parameters are based on the analysis of the pacemaker discharge of the neurons and the quantification of the AHP and interspike interval. Neurons are pooled (all) or segmented in type A and type B. Overall, the intrinsic membrane properties recorded in controls and VVM mice show limited differences between type A and type B neurons. AHPR, dAHP, and AHP are parameters representing kinetics of the AHP and interspike interval (see Materials and methods). Statistically significant differences are highlighted in red. NS, not significant.

**Fig. 6.**
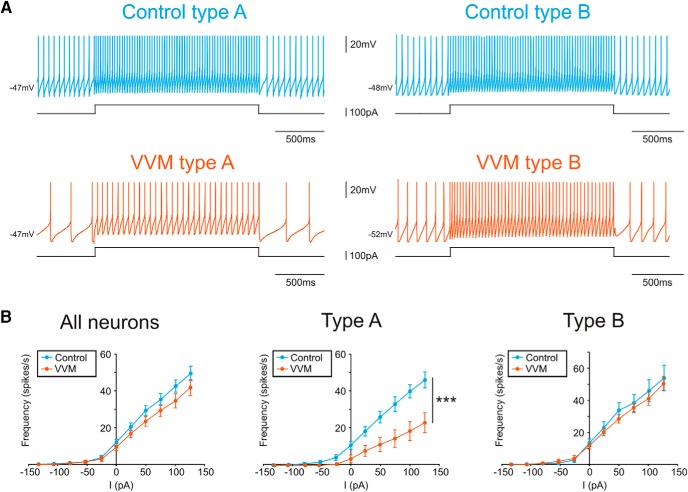
Excitability of second-order vestibular neurons in response to step-like currents. ***A***, Example raw traces of MVN neurons recorded in control (top) type A (left) and type B (right), or VVM (bottom) conditions. ***B***, Relation between the injected current and the frequency of discharge (I/F curves) for all MVN neurons (left, *n* = 38 control and *n* = 24 VVM), type A (middle, *n* = 23 control and *n* = 7 VVM) and type B (right, *n* = 15 control and *n* = 17 VVM) neurons. (***p < 0.001).

We then used step-like current stimulation (Fig. 6*A*) to investigate the excitability of the neurons by assessing their current–frequency relationship (I/F curve). No significant differences in I/F curves between control (*n* = 38) and VVM (*n* = 24) conditions were found when neuronal subtypes were pooled together (Fig. 6*B*, left; on depolarizing steps, repeated-measures ANOVA, group effect: *F*_1,52_ = 1.03, *p* = 0.316). However, when neurons were segmented into type A and type B, we found a significant decrease in the excitability restricted to type A neurons (*n* = 23 control, *n* = 7 VVM; Fig. 6*B*, middle; on depolarizing steps, repeated-measures ANOVA, group effect: *F*_1,26_ = 8.00, *p* = 0.009; group × current interaction: *F*_5,130_ = 4.88, *p* = 0.0004), whereas VVM had no significant effect on the excitability of type B neurons (*n* = 15 control, *n* = 17 VVM; Fig. 6*B*, right; on depolarizing steps, repeated-measures ANOVA, group effect: *F*_1,24_ = 0.02, *p* = 0.88). We then calculated the resistance of the neurons using hyperpolarizing steps and found that the resistance of both type A neurons and type B was not different in control and VVM neurons (type A control 310 ± 33 vs. type A VVM 302 ± 46 MΩ; type B control 382 ± 54 vs. type B VVM 370 ± 47 MΩ; Mann–Whitney test, *z* = –0.11 for type A, *z* = 1.10 for type B, not significant).

Overall, these *in vitro* results demonstrate that after 14 d of VVM, long-term reduction of the VOR is accompanied by changes occurring in the brainstem, within the direct VOR pathway. These changes consist of both a reduction of the efficacy of the vestibular afferents synapses on MVN neurons and changes in the intrinsic membrane properties of some MVN neurons.

## Discussion

Using VVM in freely behaving mice, we showed neural evidence that long-term VOR reduction is correlated to plasticity within the vestibular nuclei.

### A new protocol for VOR reduction through visuo-vestibular mismatch

In humans and monkeys, VOR adaptation is studied by the use of prisms that the subject wears for several days (Berthoz et al., 1981; Melvill Jones et al., 1988; Anzai et al., 2010; Nagao et al., 2013). On the other hand, traditional protocols triggering a VOR gain-down adaptation in rodents involve rotating the head-fixed animal in phase with the visual surround. This basic procedure is then repeated on several consecutive days to drive long-term adaptation (Raymond and Lisberger, 1996; Boyden and Raymond, 2003; Rinaldi et al., 2013). This methodology is time-consuming and consists of discontinuous training sessions interrupted by intertrial intervals of variable duration (Boyden and Raymond, 2003). It furthermore represents passive learning, since the vestibular stimulation is not actively generated by voluntary movements (Roy and Cullen, 2004; Cullen, 2012). VVM methodology bypasses these experimental constraints. Here, the VOR reduction occurs in response to voluntary natural head movements in an uninterrupted process. Using this approach, we report a general reduction of VOR by ∼50%. Our data on open-field locomotion show that mice adapt to the device and that the animals ambulate and explore the environment relatively normally, with no indication of a generalized vestibular impairment. The results obtained on sham animals further demonstrate that the reduction of VOR is a consequence of the visuo-vestibular mismatch and not of a generalized motor impairment. This decrease is comparable to the ones observed using standard VOR gain-down adaptation protocols in head-fixed animals (Boyden et al., 2004; Kassardjian et al., 2005). Moreover, VVM was shown to differentially affect the timing (phase) of the VOR depending on the tested frequency. This result can be interpreted in the framework of frequency-selective channels for vestibular processing (Baker et al., 1981; Lisberger et al., 1983; Straka et al., 2009), and of phase crossover after classic VOR adaptation. Phase crossover after gain-down learning induces phase lags at frequencies below the training frequency and phase leads at frequencies above the training frequency (Lisberger et al., 1983; Raymond and Lisberger, 1996). We show that VVM induces a similar effect on the timing of eye movements. The crossover occurs between 0.2 and 0.5 Hz, which is compatible with the natural range of mouse head movements dominated by low frequencies (Beraneck et al., 2008). Frequencies <0.5 Hz also correspond to the range at which the visual information is most important for gaze stabilization (Faulstich et al., 2006). As the changes in eye movements after VVM are comparable to those observed after a standard head-fixed protocol driving gain down, VOR reduction observed in both cases could depend on comparable cellular mechanisms. We note, however, that the VVM constitutes a different protocol from the classic VOR adaptation performed in mice, and that caution should be taken when interpreting results obtained under different experimental conditions.

### The role of brainstem changes in long-term VOR reduction

The hypothesis of a transfer of memory from the cerebellum to downstream structures has been explored using different types of cerebellar-dependent motor learning. Eyelid conditioning protocols have demonstrated that the initiation of learning in the cerebellar cortex (Ohyama and Mauk, 2001) is followed by the induction of plasticity in downstream nuclei for consolidation (Kleim et al., 2002; Ohyama et al., 2006). A comparable sequence of memory formation was demonstrated using optokinetic gain-up learning (Shutoh et al., 2006; Okamoto et al., 2011). OKR studies also suggested that long-term motor learning could depend on plastic processes within the vestibular nuclei (Shutoh et al. 2006). Adaptation of the VOR has been proposed early on to depend on several sites of plasticity in the cerebellum and the brainstem (Lisberger et al., 1981, 1994; Broussard and Lisberger, 1992; Pastor et al., 1994; du Lac et al., 1995; Dietrich and Straka, 2016). These experimental results have inspired recent theoretical studies that suggested that memory consolidation of VOR motor learning occurs in the vestibular nuclei. Here, we provide evidence of plasticity in vestibular nuclei after a long-term reduction of the VOR. The decrease of synaptic efficacy at the level of vestibular afferent synapses onto vestibular nuclei neurons is in direct line with theoretical predictions regarding VOR gain-down adaptation (Masuda and Amari, 2008; Menzies et al., 2010; Yamazaki et al., 2015). Moreover, it is consistent with a recent study showing that a change at this synapse is sufficient to induce a persistent decrease of the VOR *in vivo* (Mitchell et al., 2016). This brainstem memory raises several fundamental questions, including which subpopulations of neurons are concerned and which cellular mechanisms underlie these plastic changes.

### The majority of MVN neurons are floccular target neurons

MVN neurons that receive inputs from the flocculus are named floccular target neurons (FTNs). FTNs integrate cerebellar and vestibular inputs and are key players in VOR modulation. Based on *in vivo* studies, it was proposed that two different pathways, both projecting to ocular motoneurons, would mediate the VOR: the modifiable pathway composed of FTNs and the unmodifiable pathway composed of non-FTNs (Broussard and Kassardjian, 2004). Early *in vitro* electrophysiology suggested that a relatively low proportion of MVNs are FTNs (<15%; Babalian and Vidal 2000; Sekirnjak et al. 2003). Importantly, recent anatomical studies performed on mice demonstrated that the majority of MVNs are actually FTNs (∼80%; Shin et al. 2011), which segregate in subpopulations according to the amount (dense vs. sparse) and location (somatic vs. dendritic) of cerebellar inputs received as well as their neurotransmitter content (glutamatergic, glycinergic, GABAergic; see Fig. 1; Shin et al., 2011; Matsuno et al., 2016).

Here, because FTNs were not specifically recorded, the reduction of synaptic efficacy represents the average decrease found in the entire population of second-order MVNs likely composed of densely and sparsely innervated FTNs, as well as non-FTN neurons. In line with the possibility of a widespread change in vestibular pathway, Shutoh et al. (2006) have reported an increase in the vestibular field potential, suggesting that the OKR gain-up long-term adaptation similarly concerned a majority of the vestibular neurons *in vivo*. Regardless of the actual proportion of MVN neurons receiving monosynaptic floccular inputs, cerebellar regulation of vestibular activity could, in the long-term, spread to non-FTNs through local networks. Shin et al. (2011) reported that about half of commissural neurons are sparsely contacted by FTNs, demonstrating the influence of cerebellar inputs on the regulation of bilateral vestibular activity beyond the first synaptic contact. This hypothesis is further considered below.

### Synaptic plasticity: cellular and molecular mechanisms

The role of the input of Purkinje cells (PCs) in the induction of plasticity in the brainstem is well supported (Wulff et al., 2009; Zheng and Raman, 2010; Okamoto et al., 2011). How could PCs activity affect vestibular processing in FTNs? We demonstrated in this study that long-term VOR reduction is associated with a decrease in efficacy of the vestibular nerve synapses on MVNs, presumably through LTD-like mechanisms. It was shown that plasticity at this synapse can be induced by high-frequency stimulation of vestibular afferents, and that the direction of the plasticity is dependent on both developmental stage (Puyal et al., 2003) and stimulation pattern (Scarduzio et al., 2012) at basic potential (Idoux, 2015). Moreover, this plasticity is also dependent on the postsynaptic membrane potential (Pugh and Raman, 2006; McElvain et al., 2010). These *in vitro* electrophysiological data suggest that PC inhibition could guide the strengthening or weakening of vestibular nerve synapses on MVNs. The depression we report after long-term VOR reduction could be explained by a mechanism of heterosynaptic plasticity. It was theorized that this plastic process would use an anti-Hebbian input spike-timing dependent plasticity (iSTDP) mechanism, resulting from the simultaneous vestibular afferent activity and membrane hyperpolarization by PC inhibition (Menzies et al., 2010). In line with this hypothesis, a recent study demonstrated that in mouse parvocellular MVNs, inhibitory synapses from the flocculus and excitatory synapses from the vestibular nerve axons are often colocalized on distal dendrites of FTNs (Matsuno et al., 2016). Notably, the iSTDP mechanism could in theory also regulate the interaction of vestibular inputs with other, nonfloccular inhibitory inputs. In particular, the demonstration of a commissural feed-forward inhibition (Biesdorf et al. 2008; Malinvaud et al. 2010) interleaved with cerebellar inputs (Shin et al. 2011) raises the possibility of a long-term homeostatic activity-dependent regulation of vestibular synapses strengthened by local and commissural GABAergic and glycinergic neurons (Bagnall et al., 2007; Biesdorf et al., 2008; see discussion in Menzies et al. 2010; Mitchell et al. 2016). What would be the molecular mechanisms underlying the reported synaptic plasticity? The decrease in synaptic efficacy could depend on postsynaptic alterations, with changes in the glutamatergic receptors. In support of this hypothesis, it has been demonstrated that LTD at vestibular nerve synapses on vestibular nucleus neurons depends on NMDA receptors in mice (McElvain et al., 2010; Menzies et al., 2010). Because no additional decrease can be triggered in neurons from VVM mice using standard LTD protocols, NMDA receptors are likely a major player in the plastic process. Additional work will be needed to specify the molecular mechanisms involved in this long-term synaptic plasticity.

### Floccular target neurons comprise both type A and type B neurons

Based on electrophysiological criteria, MVN neurons are composed of at least two subpopulations (Serafin et al., 1991). The classification used in the present study identifies type A and type B neurons based on the spike AHP and shape of the interspike interval (Beraneck et al., 2003). How are type A and type B neurons inserted into vestibular-related networks? Both types receive direct excitatory vestibular inputs (Babalian et al., 1997; Pettorossi et al., 2011) and commissural inhibition (Camp et al., 2006). The majority of type A are GABAergic neurons (Takazawa et al., 2004; Bagnall et al., 2007), which receive mostly GABAergic inhibitory inputs (Camp et al., 2006). Type A neurons likely represent the majority of local interneurons (Takazawa et al., 2004) and a significant proportion of the sparse FTNs that participate in the feed-forward local regulation of MVN activity (Biesdorf et al., 2008; Malinvaud et al., 2010; Shin et al., 2011).

On the other hand, type B neurons are glutamatergic or glycinergic output neurons that project to the ocular motor nuclei (Beraneck and Idoux, 2012). Early *in vitro* studies reported that FTNs show membrane properties specific to a subset of type B neurons (Babalian and Vidal, 2000; Sekirnjak et al., 2003). This subpopulation of FTNs with highly nonlinear properties likely corresponds to densely innervated glycinergic neurons (∼10%; Shin et al., 2011; Kodama et al., 2012). In addition, the majority of glutamatergic FTNs, which project axons to the ocular motor nuclei, and of glycinergic neurons, which project to the contralateral side, are also likely to be type B neurons (Bagnall et al., 2007; Shin et al., 2011). Overall, available data suggest that FTNs are composed of both type A and type B neurons that are differentially inserted within vestibular networks and play functionally distinct roles.

In this study, the decrease of eEPSC amplitude was found on a population composed of both type A and type B neurons: the mean eEPSC amplitude was reduced by ∼50% in type A and ∼35% in type B. Although our available data do not allow for a definitive conclusion, they suggest that both populations are susceptible to show synaptic plasticity after VVM, and we have therefore no evidence for a differential implication of these subpopulations in the reported synaptic plasticity.

### Differential change in the intrinsic properties of type A and type B neurons

Reorganization within the vestibular pathway has been extensively studied in the context of postlesional modifications (i.e., vestibular compensation; Curthoys, 2000; Straka et al., 2005). In addition to synaptic plasticity (Vibert et al., 2000; Grassi and Pettorossi, 2001), changes in the intrinsic membrane properties of central vestibular neurons occur over the long time scale of several weeks, with differential changes in type A and type B neurons (Him and Dutia, 2001; Beraneck et al., 2003, 2004). Changes in the intrinsic excitability of MVNs were already postulated as a putative mechanism after VOR learning (du Lac, 1996; Broussard and Kassardjian, 2004; Gittis and du Lac, 2006). Pettorossi et al. (2011) demonstrated on brainstem slices that high-frequency stimulation of vestibular afferents leads to differential synaptic and intrinsic plasticity in type A or type B neurons, respectively. In particular, changes in intrinsic excitability were more consistently triggered in type A neurons than in type B neurons. Here, we report a decrease in the spontaneous discharge and the intrinsic excitability of type A MVNs. Although the electrophysiological classification we use does not differentiate the heterogeneous populations of type A neurons (Kodama et al., 2012), it identifies inhibitory GABAergic neurons as a key component in the tuning of the direct vestibular pathway following long-term VOR reduction. The precise identification of the different subpopulations of MVNs using, for instance, genetically engineered mice (Bagnall et al., 2007; Kodama et al., 2012) or tracing techniques (Sekirnjak and du Lac, 2006; Matsuno et al., 2016) will be the next step in understanding the cellular mechanisms involved in VOR long-term reduction after a visual-vestibular mismatch.

## Synthesis

Reviewing Editor: Leonard Maler, University of Ottawa Decisions are customarily a result of the Reviewing Editor and the peer reviewers coming together and discussing their recommendations until a consensus is reached. When revisions are invited, a fact-based synthesis statement explaining their decision and outlining what is needed to prepare a revision will be listed below. The following reviewer(s) agreed to reveal their identity: Hans Straka.

Dear authors,

Your Ms has now been carefully reviewed. The reviews are consistent. This Ms is potentially suitable for publication but does require substantial revisions and re-review. Please be sure to carefully address all the points raised by the two reviewers.

Reviewer #1

The combination of experiments (in vivo and in vitro physiology) provides a novel approach and yielded results that otherwise would not have been able to obtain. Furthermore, the demonstration of clear changes at the level of the vestibular nuclei provides evidence that the signaling in the direct VOR pathway has been altered.

This manuscript shows that the long-term reduction of the VOR after visuo-vestibular mismatch training is potentially caused by changes in the synaptic transmission from vestibular afferents onto central vestibular neurons and alterations of intrinsic properties of the latter. In a first set of experiments, the authors used a new protocol in which mice were exposed to a constant visuo-vestibular mismatch for two weeks, inducing a 50% reduction of the VOR gain. Pharmacological inactivation of the bilateral flocculus/paraflocculus indicated that the VOR reduction depends on changes outside the cerebellum. In a second set of experiments the authors used brainstem slices for recordings of MVN neurons. EPSCs in patch-clamp recorded neurons were activated by electrical stimulation of the central fiber bundle of the vestibular nerve. The analysis showed that the evoked synaptic activity was decreased after the training compared to controls, likely due to depression-like mechanisms. These changes were supplemented by a decrease in the spontaneous discharge and excitability, mainly of type A MVN neurons. In combination, the authors conclude that longer-lasting visuo-vestibular mismatch training causes synaptic and intrinsic changes outside the cerebellum within the brainstem vestibular circuitry.

GENERAL COMMENTS

The data in the present study derive from a solid and in part novel experimental approach; the data are clear, well documented, and highly interesting for the field. There are no major drawbacks concerning the experimental procedure although, as usual there would be additional feasible experiments, which could have been done. Nonetheless, the reviewer considers it a complete story that gives enough room for sequels.

There are however a couple of points, not necessary major concerns, but issues that need to be taken into account.

1. While the manuscript generally reads well, there are a number of smaller grammatical and syntax issues that as a whole, which often block the flow during reading. Therefore, please have a native English speaking person read the text. Also, the material and method section appears to have been written by different authors and not homogenized sufficiently afterwards to appear as a single piece (see below).

2. The reviewer was surprised by the finding that mainly type A MVN neurons experienced a change in the excitability. The reason is that Babalian and Vidal 2000 showed that FTNs are type B MVN neurons, and the study by Sekirnjak et al., 2003 came to the same conclusion, provided one compares the action potential shapes. This issue has been discussed in the cited review by Straka et al., 2005. This opens the question of why cerebellar output terminates on type B neurons, but long-term changes were observed in type A neurons here. Is this a real discrepancy or can it be resolved and reconciled by logic arguments.

In any case it has to be discussed in the last two paragraphs with considerably more than a mere sentences.

SPECIFIC COMMENTS

Line 8: change to ...experiments...

Line 11: change to ...afferent...

Line 12: change: ...efficiency to efficacy... here and throughout the text.

Line 12: change: ...between the vestibular ... to ...between vestibular...

Line 18: change: ...leads to synaptic and intrinsic changes... to ...leads to changes in synaptic transmission and intrinsic properties of MVN neurons

Line 19: change: ...Overall, the study opens... to ...Overall these results open ...

Line 20: Remove: ...on the mouse model.

Line 62: change to: ...have shown...

Line 72: change to: ...analyzes...

Lines 79-86: Please add the total number used animals.

Lines 89-99: Please put all sentences in past tense.

Line 91: What is meant with the "face" of the device. Please explain and reword.

Line 98: ...in behavioral experiments or used for...: Why or? Were mice for the electrophysiological recordings not tested before for gain reduction? Please be more explicit.

Line 108: What was the procedure for sham animals, how were they treated? What was the sham procedure? Please add.

Line 116: change to ... The set-up consisted...

Line 128: change to ...allowed analyzing the mice...

Line 128: change ...walked... to ...traveled...

Line 131: change procedures in headline to performance

Line 144: change to ... procedure was similar...

Line 159: Please indicate how many were excluded and included, respectively.

Line 160: change to ...around the vertical...

Line 164: change to ...in 10 out of 13 animals...

Line 165: Please rephrase the term ...measured under Matlab environment... here and elsewhere thereafter. Analyzed in Matlab would be sufficient.

Line 171: as above. Please change to Optokinetic reflex performance and...

Line 172: ...different neural area... This is slang. Please reword.

Line 174: Please indicate here that both sides were inactivated.

Lines 186-191: How was the flocculus accessed? Please be more specific.

Line 190: change to ...darkness...

Lines 202-203: Total numbers should also be indicated below at the description of the experiments.

Line 219: change to ...neuron recordings...

Line 226: change to ...in current-clamp...

Line 228: change to ...for current...

Line 234: change to ...EPSC recordings

Line 235: Please indicate here what the sham condition is.

Line 239: ...placed on the vestibular nerve... This is incorrect. The nerve was obviously cut during the preparation and the stimulation occurred on the slice. Thus, the authors have stimulated the central fiber bundle after the entrance of the nerve into the brain. While the reviewer is no proponent of this method, because of the potential current spread, he also knows that this is the best one can get with slice preparations.

Nonetheless be more specific and rephrase.

Line 243: change to ...was set at 300-400 pA...

Line 247: change to Evoked EPSC amplitude

Line 248: change to ...For the LTD...

Line 258: change to: inter-spike interval profiles...

Line 258: What is meant with tabulated? Please rephrase.

Line 270: The table is not necessary since all values are mentioned in the text and/or figure legend.

Line 271: Change to (Analysis of variance)

Line 304: change to ...velocities from 20-50...

Line 316: change to ...affects the VOR...

Line 335: change to ...noted...

Line 337: (Figure 4A, black trace): Is this trace form a control or VVM mouse? How does this compare with the respective other condition? Please show data for control and VVM mice.

Lines 342-348: Please put all in past tense.

Lines 371-372: change to ...between vestibular afferents and central...

Line 375: there is no Fig. 5D. Please change to 5C.

Line 398: Please reword "impacted" and change ...weaker firing rate... to ...lower firing rate...

Line 400: change to ...used step-like current stimulation...

Lines 413-416: Can the changes in Fig. 5 also be distinguished by subdividing the recorded neurons into type A and B? If yes, please add. If no (maybe because for technical reasons) please add a comment. Also add a short comment here and a longer one in the discussion on the issue of type B FTN neurons.

Line 423-450: if there is a word restriction for the discussion, the authors should shorten this part to give more space for the discrepancy on FTNs (see below).

Line 437: change to ...affect the...

Line 450: change to ...obtained under different...

Line 461: see also Dietrich and Straka, 2016.

Line 464: change to ...in the vestibular...

Line 473-492: The reviewer wonders why in the description of FTNs did not mention their identity as type B MVN (see Babalian and Vidal, 2000; Sekirnjak et al., 2003). This should definitely be added here in this paragraph. Also, didn´t Lisberger promote the idea that the phasic pathway (and thus more type B-like pathways) is the modifiable? See also the lengthy discussion in Straka et al., 2005 on this issue.

Thus, in this paragraph the authors should add the apparent/obvious discrepancy of the fact that FTNs are type B MVN neurons while the changes were observed in type A neurons.

Line 503-508: Please modify in view of the fact that FTNs are type B neurons.

Line 711-808: The figure legends are generally to long, wordy and descriptive and thus to a large extent repetitive with the result section. Please shorten and reduce to the description on what is shown in A,B....

Line 811: Table 1 is not necessary ad can this be removed. All is emphasized in the text already and nobody will consult the table.

All figures are very clear and all necessary issues (except the one in 4A) are illustrated.

Reviewer #2

In this manuscript the authors use a helmet-like device to induce plastic changes in the vestibulo-ocular reflex (VOR) of freely moving mice over prolonged periods of time. The results clearly show a reduction of the VOR gain. Electrophysiological recordings from brainstem slices also reveal synaptic changes in neurons located at the medial vestibular nucleus (MVN). The experiments and the results are interesting, and the overall approach is promising for inducing naturalistic plasticity.

Statistics

I did not thoroughly critique the statistics. There were some places (mentioned in my review) where the n's were almost certainly too low to demonstrate the lack of significance that is claimed.

In this manuscript the authors use a helmet-like device to induce plastic changes in the vestibulo-ocular reflex (VOR) of freely moving mice over prolonged periods of time. The results clearly show a reduction of the VOR gain. Electrophysiological recordings from brainstem slices also reveal synaptic changes in neurons located at the medial vestibular nucleus (MVN). While the experiments and the results are interesting, and the overall approach is promising for inducing naturalistic plasticity, at present the authors have not sufficiently demonstrated that the results they observe, on either a behavioral or an electrophysiological level, in fact result from visuo-vestibular mismatches, as is claimed throughout the paper. Results from critical control experiments and/or significant toning-down of the conclusions are necessary.

Major Comments

1. The authors argue that the results presented are a consequence of mismatches between visual and vestibular information, but with the data available it is unclear whether this is actually the case. Abnormal head movements produced by the extra-weight of the helmet, and/or the restricted field of view induced by the helmet itself, could lead to overall vestibular suppression and lead to the same results. To address this the authors need to show data from appropriate control animals, ideally mice that have been subjected to the same helmet wearing but without visual manipulations. In fact, the authors do mention that they have used sham animals with helmets upside-down (line 96), but those controls appear to only be used for the weight comparisons in Fig. 2 and a few neural recordings (line 235). If the authors want to claim that their results have anything to do with visuo-vestibular mismatch, they need to demonstrate that with the appropriate controls.

2. The authors focus on gaze stabilization and particularly VOR gain. However because of the concerns raised in point 1, above, it is not clear that the vestibular plasticity reported here is specific to gaze or eye movements. If the VOR gain reduction is due to overall vestibular suppression, then vestibulospinal reflexes could be affected, for example. This might even explain why they are able to observe such dramatic physiological effects at what surely must be vestibular afferent-brainstem synapses that are controlling much more than VOR gain. It would seem more appropriate to refer to the VOR gain reduction effects shown here as one readout of vestibular suppression that their paradigm invokes, rather than attempting to portray their manipulation as one that specifically affects VOR gain.

3. The negative results obtained in the flocculus shutdown experiment are not convincing. n=2 saline controls is too low to conclude that OKR gain is not significantly affected in this condition. What does flocculus shutdown do to control animals? The lidocaine condition does appear to differ from the VVM condition at higher frequencies, and as presented we cannot distinguish between lidocaine inducing a generalized suppression of VOR gain (which it might do even in controls) vs. not affecting the reduced gain following VVM as the authors suggest.

Minor Comments

1. The manuscript should be edited by a native English speaker, there are a number of mistakes in the abstract alone.

2. Line 39 OKR and VOR cooperate to stabilize the visual scene in response to self-motion, yes, but VOR is not required for external movement only

3. Line 47 Yang and Lisberger 2014 is about smooth pursuit learning, not VOR adaptation.

4. Line 71 and elsewhere, vestibular plasticity would be a more appropriate term than VOR plasticity (for the reasons outlined above)

5. Fig. 2B legend should read "sham," not "Before VVM"

6. Line 97: VVM acronym used first time without prior description

7. Line 138: "10 min before [to] start"

8. Note that the small decrease in OKR gain in Fig. 4A following VVM, although not significant, is the opposite of previously described effects of VOR adaptation on OKR gain in mice (Faulstich et al). Could this suggest that the VOR reduction shown here is in fact generalized suppression rather than VOR gain reduction per se?

9. Lines 150 and 375: Incorrect reference to nonexistent Figure 5D

10. Line 235 - Four neurons from the sham control condition is very few to be confident about pooling these results with other controls). Line 732, "control mice" makes it sound like this is a second group of mice, but it is actually the same mice before VVM, as explained in lines 734-735.

11. Lines 414, 498 - "achieved through" and "induces" have not been shown. "Accompanied by" or similar would be more appropriate.

12. Fig 3C, left: x-axis label is missing
